# Relationship Agreement Between Demirjian Tooth Development and Cervical Vertebral Maturation in Thai Children and Adolescents

**DOI:** 10.3390/jcm15083079

**Published:** 2026-04-17

**Authors:** Suttiwat Jeamtrakool, Phuwadon Duangto, Pennipat Nabheerong, Chairat Charoemratrote, Pornpat Theerasopon

**Affiliations:** 1 Department of Radiology, School of Medicine, University of Phayao, Phayao 56000, Thailand; suttiwat.je@up.ac.th (S.J.); pennipat.na@up.ac.th (P.N.); 2 Division of Anatomy, School of Medical Sciences, University of Phayao, Phayao 56000, Thailand; pete_anatomy@hotmail.com; 3 Department of Preventive Dentistry, Faculty of Dentistry, Prince of Songkla University, Songkhla 90112, Thailand; metalbracket@outlook.co.th; 4 Department of Orthodontics, School of Dentistry, University of Phayao, Phayao 56000, Thailand

**Keywords:** cervical vertebral maturation, lateral cephalometric radiograph, panoramic radiograph, Thai population, tooth development

## Abstract

**Background/Objectives**: The growth status of children and adolescents with discrepancies of maxilla/mandible during the growing period should be closely monitored to determine the appropriate time to begin growth modification in orthodontic treatment. Skeletal growth assessment using the cervical vertebral maturation (CVM) method is widely used and accepted; however, monitoring requires additional doses of radiation. Thus, this study aimed to evaluate tooth development from routine panoramic radiographs to represent the growth status rather than using the CVM method. **Methods**: Three hundred and sixty pairs of lateral cephalometric and panoramic radiographs (180 males and 180 females) aged 7–15 years were included. Teeth 31–37 of each panoramic radiograph were identified as A to H according to the Demirjian method, and the stages of skeletal growth were indicated from lateral cephalometric radiographs using the CVM method. The relationship between tooth development and CVM was analyzed using Spearman’s rank correlation coefficients. **Results**: The correlation coefficients ranged from 0.487 to 0.768 for male subjects and from 0.503 to 0.759 for female subjects. Tooth 33 was found to have the highest correlation in males (r = 0.768) and tooth 37 was revealed to have the highest correlation in females (r = 0.759) (*p* < 0.01). **Conclusions**: Teeth 33–37 showed correlation coefficients close to 0.7 or above, which indicated a moderate-to-high correlation between tooth development and CVM. Thus, the pattern of tooth development from teeth 33–37 may serve as a supplementary indicator of skeletal maturation timing which was similar in both males and females, and may serve as a supplementary indicator of skeletal maturation timing.

## 1. Introduction

Individual skeletal maturity assessment is known as the most precise method, as opposed to other methods such as the use of chronological age or dental age, to evaluate biological age to predict the growth status of the maxilla and mandible. Two evidence-based radiographic methods that have been used include hand and wrist radiographs and lateral cephalometric radiographs. Previous studies showed a high correlation between these two methods and have been routinely used in clinical orthodontic practice [1,2]. However, hand and wrist radiographs require more exposure to radiation, which has led to the recommendation that hand and wrist radiographs be excluded from the guideline [3,4,5]. Therefore, the cervical vertebral maturation (CVM) method using lateral cephalometric radiographs for skeletal growth evaluation has become more popular.

CVM is one of the prediction schemes of skeletal maturity indices to assess the cross-sectional observation of skeletal development in children and adolescents. In 2005, Baccetti and colleagues published the CVM index, which can be evaluated from routine lateral cephalometric radiographs to assess pubertal growth, which is highly related to jaw development [5].

In orthodontic treatment, it is crucial to determine the growth period of the maxilla and mandible when evaluating jaw discrepancies for a precise diagnosis, orthodontic treatment planning, and setting the timing to start growth modification therapy [6]. Although the CVM index from a lateral cephalometric radiograph is recommended as a routine radiograph in orthodontic practice, the benefits may be outweighed by frequent radiographs before treatment begins. A panoramic radiograph is a common routine dental radiograph that can be taken up to every six months since the dose of radiation is up to half the dose of a lateral cephalometric radiograph [7]. Furthermore, a panoramic radiograph can screen several structures in the facial area that include the bony structures of the maxillae and tooth development. The stages of tooth development were developed in 1973 by Demirjian et al. [8], who reported that panoramic radiographs were proven to be reproducible and provided perfect discrimination when assessed by healthcare staff and students [9].

Previous studies [10,11,12,13,14,15,16,17,18,19,20,21,22,23,24,25,26,27,28,29,30,31,32] found moderate to high correlations not only between Demirjian tooth development and CVM but also for ethnicity and sex. Thus, this study aimed to assess the correlation between Demirjian tooth development and CVM specifically based on a population in southern Thailand, which to our knowledge has never been reported. If the correlation is sufficiently strong enough between tooth development and the growth period, panoramic radiographs might become a screening tool to determine the level of skeletal maturity.

## 2. Materials and Methods

The cross-sectional study was approved by the Human Research Ethics Committee of the Faculty of Dentistry, Prince of Songkla University, Songkhla, Thailand (EC6810-049). Digital panoramic and lateral cephalometric radiographs, which were collected between 2015 and 2025, were retrieved from the institutional archives and stored in JPEG format using the GXDP-700 PANOREX with a cone beam unit (Gendex Dental Systems, Hatfield, PA, USA).

A total of 360 digital radiographs (180 males and 180 females) from individuals aged 7 to 15 years, who were registered in the southern region of Thailand, were randomly selected for inclusion in this study. Radiographs were excluded if they were of poor quality, demonstrated missing or extracted teeth on panoramic images, or were obtained from patients with a history of, or ongoing, orthodontic treatment. Demographic data, which included sex, date of birth, and date of radiographic acquisition, were recorded confidentially by a board-certified orthodontist (P.T.). Chronological age was calculated by subtracting the date of birth from the date of the radiograph and was expressed in years to two decimal places.

The eight stages of tooth development were labeled A through H according to the Demirjian method [8]. The stages were assessed using digital panoramic radiographs of the mandibular left teeth that included the central incisor (tooth 31), lateral incisor (tooth 32), canine (tooth 33), first premolar (tooth 34), second premolar (tooth 35), first molar (tooth 36), and second molar (tooth 37) (Table 1). Evaluation of CVM using lateral cephalometric radiographs was classified into six stages (CVM 1 to 6) based on the Baccetti method [5] (Table 2). Each stage of dental and skeletal development was independently assessed by two board-certified radiologists (S.J. and P.N.).

Data were analyzed using SPSS Statistics for Windows, version 27.0 (IBM Inc., Chicago, IL, USA) by a co-investigator (P.D.). Descriptive statistics that included mean, standard deviation, minimum, and maximum values were calculated for samples stratified by CVM stages. The analyses were performed separately for males and females. Spearman’s rank correlation coefficients were employed to evaluate the association between dental development, as assessed by the Demirjian method, and skeletal maturation based on the Baccetti CVM method, with sex-specific analyses conducted. A significance level of 0.01 was adopted. Intra-observer reliability was assessed by randomly selecting 40 panoramic and 40 lateral cephalometric radiographs, which were re-evaluated one month after the initial assessment by the same radiologist, who was blinded to prior results. Inter-observer agreement was further examined between two radiologists using 40 radiographs. The level of agreement was determined using the weighted kappa statistic.

## 3. Results

The distribution of the study subjects according to the CVM method is presented in Table 3. The mean chronological ages for each period of skeletal maturity were consistently higher in male subjects. The mean chronological age of the male group was older than that of the female group by approximately 1.28 years (range, 0.48 years through 1.72 years). The reproducibility of all assessments was found to be almost perfect with high values. Weighted Kappa values of intra-observer agreement were 1.000 for the CVM and 0.981–1.000 for the tooth assessment. The inter-observer agreement was found to be 0.975 for the CVM and 0.925–0.989 for tooth assessment (Table 4).

The relationships between the skeletal maturity stages from the lateral cephalometric radiograph and tooth development of the seven mandibular teeth (teeth 31–37) are presented in Table 5. Spearman’s rank correlation coefficients (r) and their corresponding 95% confidence intervals (95% CI) were calculated for both male and female subjects.

In males, the correlation coefficients ranged from 0.487 to 0.768, while in females they ranged from 0.503 to 0.759. Tooth 33 demonstrated the highest correlation in males (r = 0.768, 95% CI: 0.698–0.823), whereas tooth 37 showed the highest correlation in females (r = 0.759, 95% CI: 0.687–0.816).

Conversely, tooth 31 exhibited the lowest correlation in both sexes (males: r = 0.487, 95% CI: 0.363–0.594; females: r = 0.503, 95% CI: 0.381–0.607). All correlations were statistically significant (*p* < 0.01).

The percentage distributions for the relationship between the Demirjian method of individual teeth in the timing of pubertal growth using the CVM method are shown in Table 6, Table 7, Table 8, Table 9, Table 10 and Table 11. For each of the stages, only the highest proportion was selected for representation.

At the pre-peak stage (CVM 1–2) in male subjects, teeth 31, 32, and 36 had the highest percentages in stage H, which were 59.34%, 47.25%, and 57.14%, respectively. Teeth 33, 34, and 35 had the highest percentages in stage F (64.84%, 39.56%, and 39.56%). Finally, tooth 37 had the highest percentage in stage E (28.57%) (Table 6). In female subjects, teeth 31, 32, and 36 also had the highest percentages in stage H, which were 49.35%, 37.66%, and 48.05%, respectively. Teeth 33, 34, and 35 also had the highest percentages in stage F (66.23%, 53.25%, and 46.75%), while tooth 37 had the highest percentages in stages D and E, which were 32.47% equally (Table 9).

At the peak stage (CVM 3–4) in male subjects, teeth 31–36 had the highest percentages in stage H (98.44%, 95.31%, 43.75%, 68.75%, 43.75%, and 100.00%), while tooth 37 had the highest percentage in stage G (40.63%) (Table 7). Likewise, in female subjects, teeth 31–36 had the highest percentages in stage H (91.80%, 93.44%, 47.54%, 60.66%, 36.07%, and 98.36%), whereas tooth 37 had the highest percentage in stage G (40.98%) (Table 10).

At the post-peak stage (CVM 5–6), all teeth had the highest percentages in stage H in both males (100.00%, 96.00%, 80.00%, 96.00%, 96.00%, 100.00%, and 52.00%) (Table 8) and females (97.62%, 90.48%, 66.67%, 78.57%, 57.14%, 100.00%, and 47.62%) (Table 11).

## 4. Discussion

### 4.1. Interpretation

The present study found that the mean chronological age in females was lower than that of males in all CVM stages (Table 3), which indicated that females reach each CVM stage earlier than males. This finding is consistent with previous studies [14,15,16,17,18,19,21,22,24,25,26,27,30,33,34].

The weighted kappa values (Table 4) for CVM assessment using the Baccetti method and dental development assessment using the Demirjian method demonstrated almost perfect agreement. The intra-observer agreement ranged from 0.981 to 1.000, while inter-observer agreement ranged from 0.925 to 0.989, which indicated that both methods are highly reliable and repeatable within and between the observers. A major advantage of the Demirjian and Baccetti methods is the use of clear stage definitions together with illustrative reference images, which facilitate consistent interpretation and help reduce observer variability.

The strongest correlation with CVM stages in Thai males was observed for tooth 33 (r = 0.768), followed by tooth 35 (r = 0.762), tooth 34 (r = 0.754), and tooth 37 (r = 0.743). In Thai females, tooth 37 showed the strongest correlation (r = 0.759) followed by tooth 34 (r = 0.730), tooth 35 (r = 0.702), and tooth 33 (r = 0.677). The findings indicated that the same four teeth (33, 34, 35, and 37) demonstrated moderate-to-high correlations (r = 0.5–0.7 moderate, >0.7 strong, depending on clinical context) with the CVM stages in both Thai males and females, while the rank order varied between sexes.

### 4.2. Comparison with Previous Literature

The strongest correlation with CVM stages was observed for tooth 33 in males and tooth 37 in females. These findings are consistent with previous studies in Asian populations that included Indian [28,34] and Chinese populations [18], as well as European populations such as white British subjects [22].

In males, a previous study similarly reported that tooth 33 had the highest correlation to CVM stages in European populations such as Romanian groups [30].

In females, several studies have identified tooth 37 as having the strongest correlation with the CVM stages. This finding was also reported in Asian populations [22], which included the Korean [33], Tunisian [19], Yemeni [14], Iranian [27,29], North Karnataka [15], and Indian populations [16,20], as well as in European populations, such as French [17] and Greek subjects [24]. Additional supporting evidence is presented in Table 12.

The CVM method for evaluating pubertal growth is divided into three groups: CVM 1–2 is the pre-peak stage, CVM 3–4 is the peak stage, and CVM 5–6 is the post-peak stage according to Baccetti et al., 2005 [5].

In males at the pre-peak stage (CVM 1–2), the highest percentage in each tooth were as follows: tooth 31-stage H (59.34%), tooth 32-stage H (47.25%), tooth 33-stage F (64.84%), tooth 34-stage F (39.56%), tooth 35-stage F (39.56%), tooth 36-stage H (57.14%), and tooth 37-stage E (28.57%). At the peak stage (CVM 3–4), the predominant stage was H for most teeth, which included tooth 31 (98.44%), tooth 32 (95.31%), tooth 33 (43.75%), tooth 34 (68.75%), tooth 35 (43.75%), and tooth 36 (100%). However, tooth 37 mostly presented at stage G (40.63%). At the post-peak stage (CVM 5–6), stage H was most frequently observed in all evaluated teeth: tooth 31 (100%), tooth 32 (96.00%), tooth 33 (80.00%), tooth 34 (96.00%), tooth 35 (96.00%), tooth 36 (100%), and tooth 37 (52.00%).

In females at the pre-peak stage (CVM 1–2), the highest percentages in each tooth were as follows: tooth 31-stage H (49.35%), tooth 32-stage H (37.66%), tooth 33-stage F (66.23%), tooth 34-stage F (53.25%), tooth 35-stage F (46.75%), tooth 36-stage H (48.05%), and tooth 37-stage D/E (32.47%). At the peak stage (CVM 3–4), the predominant stage was H for most teeth, which included tooth 31 (91.80%), tooth 32 (93.44%), tooth 33 (47.54%), tooth 34 (60.66%), tooth 35 (36.07%), and tooth 36 (98.36%). However, tooth 37 mostly presented at stage G (40.98%). At the post-peak stage (CVM 5–6), stage H was most frequently observed in all evaluated teeth: tooth 31 (97.62%), tooth 32 (90.48%), tooth 33 (66.67%), tooth 34 (78.57%), tooth 35 (57.14%), tooth 36 (100%), and tooth 37 (47.62%).

**Figure 1:**
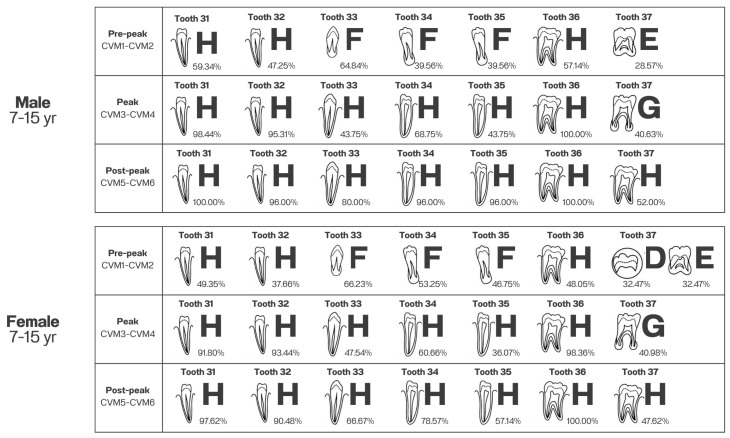
Illustration of tooth development stages to represent the timing of the pubertal growth spurt for males and females (adapted from Demirjian et al. [8]).

Figure 1 shows the highest percentage stage of each tooth classified by the timing of pubertal growth using the CVM method.

### 4.3. Clinical Implications

The findings of this study carry practical implications for orthodontic practice. Panoramic radiographs are already part of routine orthodontic records. Therefore, tooth development staging into clinical assessment represents a low-burden, radiation-conscious adjunct to a standard evaluation. Specifically, the pattern of tooth development stages observed in teeth 33–37 may alert the clinician to the likely skeletal maturation period of a patient. Thus, the timing of referral for more definitive CVM assessment or for initiating growth modification therapy can be determined. It is emphasized that this approach serves as a supplementary screening step, not as a replacement for formal CVM assessment.

An important biological context for the present findings is the relationship between CVM stages and the tooth eruption sequence. Published evidence generally indicates that peak pubertal growth (CVM 3–4) tends to coincide with specific stages of active root formation and the eruption of permanent dentition. The strong correlations observed for the canine, premolars, and second molar in this study are consistent with this biological link, as these teeth undergo active root development during the pubertal growth period. It should be noted, however, that clinical tooth eruption is influenced by additional local factors that include arch length, crowding, and extraction history, which are not captured by radiographic root development stage alone. Future studies that examine the relationship between CVM and eruption sequence directly in Thai populations would be of value.

### 4.4. Limitations

Several limitations of this study should be acknowledged. First, the cross-sectional design precludes the establishment of temporal or causal relationships between dental and skeletal maturation. Longitudinal prospective studies are required to validate the predictive utility of dental development stages for growth phase estimation. Second, the subgroup size for CVM stage 6 was small. This was a consequence of our recruitment strategy being based on chronological age rather than CVM staging. Although our primary objective was to investigate correlations during the pubertal growth spurt (pre-peak to post-peak stages), which was adequately represented, we acknowledge that this limits the generalizability of our findings for the post-pubertal period. Third, the present findings are specific to a Thai population in southern Thailand. Therefore, due to the well-documented ethnic variation in both dental and skeletal maturation, caution is required before generalizing these results to other populations. Fourth, potential confounding variables, which include nutritional status, socioeconomic conditions, and systemic health factors, were not available for adjustment due to the retrospective nature of the data. These factors may independently influence both dental and skeletal development and should be incorporated in future prospective investigations. Fifth, although Spearman’s rank correlation is the appropriate and widely used approach to assess associations of this type, it does not provide predictive capacity or diagnostic accuracy metrics. Logistic regression, ROC curve analysis, or similar frameworks would be required in future work to establish clinical decision thresholds. Finally, chronological age is closely associated with both dental and skeletal development, and the observed correlations may in part reflect this shared influence of chronological age since both dental and skeletal maturation are age-dependent processes. Therefore, part of the association observed may be driven by parallel maturation rather than a direct biological linkage, and multivariate analyses that adjust for age are recommended in future studies.

## 5. Conclusions

The present study confirms a significant correlation between tooth development and the cervical vertebral maturation (CVM) stages in a Thai population. The mandibular canine in males and the mandibular second molar in females demonstrated the strongest associations. The pattern of tooth development of teeth 33–37 on routine panoramic radiographs may serve as a supplementary clinical indicator. However, clinicians should be aware that moderate correlations may still result in clinically relevant misclassifications of growth phases, particularly when treatment timing decisions depend on precise identification of the pubertal growth spurt. Since no predictive model or diagnostic thresholds were established in this study, these findings do not support the use of panoramic dental staging as a standalone substitute for CVM evaluation from lateral cephalometric radiographs. However, skeletal growth during puberty growth may be influenced by multiple factors that include population characteristics, nutrition, genetics, environmental conditions, and socioeconomic status.

## Figures and Tables

**Table 1:** Definitions of tooth development stages according to the Demirjian method [8].

Stages	Definitions
A	“In both uniradicular and multiradicular teeth, a beginning of calcification is seen at the superior level of the crypt in the form of an inverted cone or cones. There is no fusion of these calcified points.”
B	“Fusion of the calcified points forms one or several cusps which unite to give a regularly outlined occlusal surface.”
C	“Enamel formation is complete at the occlusal surface. Its extension and convergence towards the cervical region is seen.” “The beginning of a dentinal deposit is seen.” “The outline of the pulp chamber has a curved shape at the occlusal border.”
D	“The crown formation is completed down to the cemento-enamel junction.” “The superior border of the pulp chamber in the uniradicular teeth has a definite curved form, being concave towards the cervical region. The projection of the pulp horns, if present, gives an outline shaped like an umbrella top. In molars the pulp chamber has a trapezoidal form.” “Beginning of root formation is seen in the form of a spicule.”
E	Uniradicular teeth: “The walls of the pulp chamber now form straight lines, whose continuity is broken by the presence of the pulp horn, which is larger than in the previous stage.” “The root length is less than the crown height.” Molars: “Initial formation of the radicular bifurcation is seen in the form of either a calcified point or a semi-lunar shape.” “The root length is still less than the crown height.”
F	Uniradicular teeth: “The walls of the pulp chamber now form a more or less isosceles triangle.” “The apex ends in a funnel shape.” “The root length is equal to or greater than the crown height.” Molars: “The calcified region of the bifurcation has developed further down from its semi-lunar stage to give the roots a more definite and distinct outline with funnel shaped endings.” “The root length is equal to or greater than the crown height.”
G	“The walls of the root canal are now parallel and its apical end is still partially open (Distal root on molars).”
H	“The apical end of the root canal is completely closed (Distal root on molars)” “The periodontal membrane has a uniform width around the root and the apex.”

**Table 2:** Definitions of CVM according to the Baccetti method [5].

CVM	Definitions
1	“The lower borders of all the three vertebrae (C2–C4) are flat. The bodies of both C3 and C4 are trapezoid in shape (the superior border of the vertebral body is tapered from posterior to anterior).”
2	“A concavity is present at the lower border of C2 (in four of five cases, with the remaining subjects still showing a cervical stage 1). The bodies of both C3 and C4 are still trapezoid in shape.”
3	“Concavities at the lower borders of both C2 and C3 are present. The bodies of C3 and C4 may be either trapezoid or rectangular horizontal in shape.”
4	“Concavities at the lower borders of C2, C3, and C4 now are present. The bodies of both C3 and C4 are rectangular horizontal in shape.”
5	“The concavities at the lower borders of C2, C3, and C4 still are present. At least one of the bodies of C3 and C4 is squared in shape. If not square, the body of the other cervical vertebra still is rectangular horizontal.”
6	“The concavities at the lower borders of C2, C3, and C4 still are evident. At least one of the bodies of C3 and C4 is rectangular vertical in shape. If not rectangular vertical, the body of the other cervical vertebra is squared.”

**Table 3:** Descriptive statistics of the samples grouped by CVM.

CVM	Sex	Number of Samples	Chronological Age (Years)			
			Maximum	Minimum	Mean	Standard Deviation
CVM 1	Male	65	7.23	13.86	9.42	1.57
	Female	62	7.03	12.80	9.10	1.29
CVM 2	Male	26	7.62	13.04	10.16	1.72
	Female	15	7.23	11.79	9.45	1.42
CVM 3	Male	19	7.93	13.40	11.40	1.36
	Female	16	7.90	12.81	11.10	1.30
CVM 4	Male	45	11.07	15.99	13.62	1.38
	Female	45	10.39	15.95	13.50	1.53
CVM 5	Male	22	13.48	15.97	14.70	0.72
	Female	41	11.18	15.73	13.91	1.30
CVM 6	Male	3	15.00	15.88	15.33	0.48
	Female	1	13.24	13.24	13.24	N/A

**Table 4:** Weighted kappa values of intra-observer agreement and of inter-observer agreement of CVM using the Baccetti method and dental development using the Demirjian method.

Method	Intra-Observer Agreement	Inter-Observer Agreement
CVM	1.000	0.975
Tooth 31	1.000	0.962
Tooth 32	1.000	0.925
Tooth 33	0.988	0.939
Tooth 34	1.000	0.975
Tooth 35	0.981	0.952
Tooth 36	1.000	0.951
Tooth 37	1.000	0.989

Tooth 31: left mandibular central incisor; tooth 32: left mandibular lateral incisor; tooth 33: left mandibular canine; tooth 34: left mandibular first premolar; tooth 35: left mandibular second premolar; tooth 36: left mandibular first molar; tooth 37: left mandibular second molar.

**Table 5:** Spearman’s rank correlation coefficients (r) and 95% confidence intervals (95% CI) between dental development and CVM for males and females.

Tooth	Male			Female		
	r	95% CI Lower Limit	95% CI Upper Limit	r	95% CI Lower Limit	95% CI Upper Limit
Tooth 31	0.487 **	0.363	0.594	0.503 **	0.381	0.607
Tooth 32	0.547 **	0.432	0.645	0.546 **	0.431	0.644
Tooth 33	0.768 **	0.698	0.823	0.677 **	0.586	0.751
Tooth 34	0.754 **	0.681	0.812	0.730 **	0.651	0.793
Tooth 35	0.762 **	0.690	0.818	0.702 **	0.617	0.771
Tooth 36	0.506 **	0.385	0.610	0.563 **	0.450	0.657
Tooth 37	0.743 **	0.667	0.804	0.759 **	0.687	0.816

** Correlation is significant at the 0.01 level.

**Table 6:** Percentage distribution of Demirjian stages of individual teeth at pre-peak stage (CVM 1–CVM 2) for males.

Demirjian Stages	Pre-Peak Stage (CVM 1–CVM 2)													
	Tooth 31		Tooth 32		Tooth 33		Tooth 34		Tooth 35		Tooth 36		Tooth 37	
	*n*	%	*n*	%	*n*	%	*n*	%	*n*	%	*n*	%	*n*	%
A	0	0.00	0	0.00	0	0.00	0	0.00	0	0.00	0	0.00	0	0.00
B	0	0.00	0	0.00	0	0.00	0	0.00	0	0.00	0	0.00	0	0.00
C	0	0.00	0	0.00	0	0.00	0	0.00	0	0.00	0	0.00	11	12.09
D	0	0.00	0	0.00	3	3.30	7	7.69	8	8.79	0	0.00	20	21.98
E	0	0.00	0	0.00	15	16.48	28	30.77	30	32.97	0	0.00	26	28.57
F	5	5.49	27	29.67	59	64.84	36	39.56	36	39.56	5	5.49	24	26.37
G	32	35.16	21	23.08	10	10.99	12	13.19	14	15.38	34	37.36	7	7.69
H	54	59.34	43	47.25	4	4.40	8	8.79	3	3.30	52	57.14	3	3.30
Total	91	100.00	91	100.00	91	100.00	91	100.00	91	100.00	91	100.00	91	100.00

**Table 7:** Percentage distribution of Demirjian stages of individual teeth at peak stage (CVM 3–CVM 4) for males.

Demirjian Stages	Peak Stage (CVM 3–CVM 4)													
	Tooth 31		Tooth 32		Tooth 33		Tooth 34		Tooth 35		Tooth 36		Tooth 37	
	*n*	%	*n*	%	*n*	%	*n*	%	*n*	%	*n*	%	*n*	%
A	0	0.00	0	0.00	0	0.00	0	0.00	0	0.00	0	0.00	0	0.00
B	0	0.00	0	0.00	0	0.00	0	0.00	0	0.00	0	0.00	0	0.00
C	0	0.00	0	0.00	0	0.00	0	0.00	0	0.00	0	0.00	0	0.00
D	0	0.00	0	0.00	0	0.00	0	0.00	0	0.00	0	0.00	0	0.00
E	0	0.00	0	0.00	1	1.56	1	1.56	2	3.13	0	0.00	5	7.81
F	0	0.00	1	1.56	9	14.06	7	10.94	11	17.19	0	0.00	12	18.75
G	1	1.56	2	3.13	26	40.63	12	18.75	23	35.94	0	0.00	26	40.63
H	63	98.44	61	95.31	28	43.75	44	68.75	28	43.75	64	100.00	21	32.81
Total	64	100.00	64	100.00	64	100.00	64	100.00	64	100.00	64	100.00	64	100.00

**Table 8:** Percentage distribution of Demirjian stages of individual teeth at post-peak stage (CVM 5–CVM 6) for males.

Demirjian Stages	Post-Peak Stage (CVM 5–CVM 6)													
	Tooth 31		Tooth 32		Tooth 33		Tooth 34		Tooth 35		Tooth 36		Tooth 37	
	*n*	%	*n*	%	*n*	%	*n*	%	*n*	%	*n*	%	*n*	%
A	0	0.00	0	0.00	0	0.00	0	0.00	0	0.00	0	0.00	0	0.00
B	0	0.00	0	0.00	0	0.00	0	0.00	0	0.00	0	0.00	0	0.00
C	0	0.00	0	0.00	0	0.00	0	0.00	0	0.00	0	0.00	0	0.00
D	0	0.00	0	0.00	0	0.00	0	0.00	0	0.00	0	0.00	0	0.00
E	0	0.00	0	0.00	0	0.00	0	0.00	0	0.00	0	0.00	0	0.00
F	0	0.00	0	0.00	0	0.00	0	0.00	0	0.00	0	0.00	0	0.00
G	0	0.00	1	4.00	5	20.00	1	4.00	1	4.00	0	0.00	12	48.00
H	25	100.00	24	96.00	20	80.00	24	96.00	24	96.00	25	100.00	13	52.00
Total	25	100.00	25	100.00	25	100.00	25	100.00	25	100.00	25	100.00	25	100.00

**Table 9:** Percentage distribution of Demirjian stages of individual teeth at pre-peak stage (CVM 1–CVM 2) for females.

Demirjian Stages	Pre-Peak Stage (CVM 1–CVM 2)													
	Tooth 31		Tooth 32		Tooth 33		Tooth 34		Tooth 35		Tooth 36		Tooth 37	
	*n*	%	*n*	%	*n*	%	*n*	%	*n*	%	*n*	%	*n*	%
A	0	0.00	0	0.00	0	0.00	0	0.00	0	0.00	0	0.00	0	0.00
B	0	0.00	0	0.00	0	0.00	0	0.00	0	0.00	0	0.00	0	0.00
C	0	0.00	0	0.00	0	0.00	0	0.00	0	0.00	0	0.00	5	6.49
D	0	0.00	0	0.00	1	1.30	4	5.19	5	6.49	0	0.00	25	32.47
E	0	0.00	0	0.00	8	10.39	21	27.27	26	33.77	0	0.00	25	32.47
F	3	3.90	21	27.27	51	66.23	41	53.25	36	46.75	11	14.29	17	22.08
G	36	46.75	27	35.06	11	14.29	6	7.79	8	10.39	29	37.66	4	5.19
H	38	49.35	29	37.66	6	7.79	5	6.49	2	2.60	37	48.05	1	1.30
Total	77	100.00	77	100.00	77	100.00	77	100.00	77	100.00	77	100.00	77	100.00

**Table 10:** Percentage distribution of Demirjian stages of individual teeth at peak stage (CVM 3–CVM 4) for females.

Demirjian Stages	Peak Stage (CVM 3–CVM 4)													
	Tooth 31		Tooth 32		Tooth 33		Tooth 34		Tooth 35		Tooth 36		Tooth 37	
	*n*	%	*n*	%	*n*	%	*n*	%	*n*	%	*n*	%	*n*	%
A	0	0.00	0	0.00	0	0.00	0	0.00	0	0.00	0	0.00	0	0.00
B	0	0.00	0	0.00	0	0.00	0	0.00	0	0.00	0	0.00	0	0.00
C	0	0.00	0	0.00	0	0.00	0	0.00	0	0.00	0	0.00	0	0.00
D	0	0.00	0	0.00	0	0.00	0	0.00	0	0.00	0	0.00	1	1.64
E	0	0.00	0	0.00	0	0.00	1	1.64	1	1.64	0	0.00	2	3.28
F	0	0.00	1	1.64	9	14.75	12	19.67	18	29.51	0	0.00	19	31.15
G	5	8.20	3	4.92	23	37.70	11	18.03	20	32.79	1	1.64	25	40.98
H	56	91.80	57	93.44	29	47.54	37	60.66	22	36.07	60	98.36	14	22.95
Total	61	100.00	61	100.00	61	100.00	61	100.00	61	100.00	61	100.00	61	100.00

**Table 11:** Percentage distribution of Demirjian stages of individual teeth at post-peak stage (CVM 5–CVM 6) for females.

Demirjian Stages	Post-Peak Stage (CVM 5–CVM 6)													
	Tooth 31		Tooth 32		Tooth 33		Tooth 34		Tooth 35		Tooth 36		Tooth 37	
	*n*	%	*n*	%	*n*	%	*n*	%	*n*	%	*n*	%	*n*	%
A	0	0.00	0	0.00	0	0.00	0	0.00	0	0.00	0	0.00	0	0.00
B	0	0.00	0	0.00	0	0.00	0	0.00	0	0.00	0	0.00	0	0.00
C	0	0.00	0	0.00	0	0.00	0	0.00	0	0.00	0	0.00	0	0.00
D	0	0.00	0	0.00	0	0.00	0	0.00	0	0.00	0	0.00	0	0.00
E	0	0.00	0	0.00	0	0.00	0	0.00	0	0.00	0	0.00	0	0.00
F	0	0.00	0	0.00	1	2.38	1	2.38	3	7.14	0	0.00	6	14.29
G	1	2.38	4	9.52	13	30.95	8	19.05	15	35.71	0	0.00	16	38.10
H	41	97.62	38	90.48	28	66.67	33	78.57	24	57.14	42	100.00	20	47.62
Total	42	100.00	42	100.00	42	100.00	42	100.00	42	100.00	42	100.00	42	100.00

**Table 12:** A comparison between the correlations of dental stage and CVM of this study with other studies.

Author (Year)	Population	Age Range (Year)	*n* (Male/Female)	Tooth	Correlation (r)	
					Male	Female
This study	Thai	7–15	360 (180/180)	31	0.487	0.503
				32	0.547	0.546
				33	0.768	0.677
				34	0.754	0.730
				35	0.762	0.702
				36	0.506	0.563
				37	0.743	0.759
Arora et al. (2023) [16]	Indian	8–17	270 (135/135)	31	0.063	0.151
				32	0.125	0.109
				33	0.695	0.696
				34	0.611	0.552
				35	0.727	0.602
				36	0.318	0.417
				37	0.734	0.768
Ojha et al. (2023) [28]	Indian	8–14	120 (60/60)	33	0.716	0.526
				34	0.625	0.424
				35	0.615	0.484
				37	0.652	0.556
Brotons et al. (2022) [17]	French	9–19	192 (101/91)	33	0.114	0.579
				34	0.090	0.383
				35	0.512	0.512
				37	0.618	0.618
Gmati et al. (2021) [19]	Tunisian	M 8–17 F 8–15	123 (62/61)	31	0.536	0.668
				32	0.736	0.562
				33	0.517	0.624
				34	0.664	0.764
				35	0.722	0.681
				36	0.689	0.615
				37	0.738	0.812
Kim et al. (2021) [33]	Korean	6–14	743 (359/384)	31	0.404	0.458
				32	0.576	0.573
				33	0.798	0.750
				34	0.823	0.768
				35	0.792	0.766
				36	0.611	0.575
				37	0.828	0.780
Al-Aunhomi et al. (2020) [14]	Yemeni	8–18	207 (85/122)	33	0.787	0.686
				34	0.822	0.817
				35	0.836	0.867
				37	0.871	0.873
Mauricio-Vilchez et al. (2020) [25]	Peruvian	8–17	200 (85/115)	33	0.761	0.779
				34	0.763	0.777
				35	0.650	0.784
				36	0.635	0.677
				37	0.774	0.782
Toodehzaeim et al. (2020) [31]	Iranian	8–17	125 (61/64)	37	0.805	0.908
Mollabashi et al. (2019) [27]	Iranian	8–16	600 (224/376)	31	0.222	0.218
				32	0.533	0.445
				33	0.746	0.722
				34	0.734	0.732
				35	0.693	0.718
				36	0.471	0.519
				37	0.753	0.751
Savin et al. (2019) [30]	Romanian	7–16	88 (42/46)	31	0.328	0.226
				32	0.328	0.324
				33	0.733	0.753
				34	0.651	0.742
				35	0.679	0.656
				36	0.655	0.085
				37	0.528	0.562
Lecca-Morales et al. (2017) [23]	Peruvian	7–17	78 (34/44)	33	0.578	0.542
				34	0.585	0.644
				35	0.634	0.600
				37	0.684	0.534
Mini et al. (2017) [26]	Indian	8–16	100 (46/54)	33	0.48	0.61
				34	0.51	0.69
				35	0.69	0.74
				37	0.59	0.66
Mustafa et al. (2017) [34]	Indian	8–16	60 (45/15)	33	0.33	0.29
				34	0.24	0.33
				35	0.28	0.36
				37	0.27	0.44
Hasan et al. (2016) [21]	Sudanese	7–16	112 (47/65)	31	0.453	0.276
				32	0.408	0.451
				33	0.759	0.586
				34	0.774	0.672
				35	0.752	0.772
				36	0.496	0.425
				37	0.758	0.756
Howell (2015) [22]	White British	10–18	90 (42/48)	33	0.568	0.329
				34	0.392	0.291
				35	0.565	0.469
				37	0.474	0.533
	Asian	10–18	90 (40/50)	33	0.669	0.489
				34	0.687	0.479
				35	0.696	0.526
				37	0.752	0.569
Litsas et al. (2016) [24]	Greek	8–18	255 (110/145)	33	0.49	0.53
				34	0.59	0.64
				35	0.59	0.63
				37	0.65	0.72
Ravadgar et al. (2015) [29]	Iranian	8–16	216 (99/117)	33	0.67	0.76
				34	0.70	0.76
				35	0.71	0.76
				37	0.63	0.77
Torun and Oktay (2015) [32]	Turkish	7–18	200 (100/100)	33	0.538	0.538
				34	0.522	0.475
				35	0.557	0.530
				37	0.501	0.462
Ara et al. (2014) [15]	North Karnataka	7–16	50 (24/26)	33	0.461	0.403
				34	0.496	0.452
				35	0.512	0.508
				37	0.491	0.531
Valizadeh et al. (2012) [12]	Iranian	8–14	400 (-/400)	31	-	0.71
				32	-	0.47
				33	-	0.73
				34	-	0.75
				35	-	0.71
				36	-	0.37
				37	-	0.34
Gupta et al. (2011) [20]	Indian	8–14	100 (59/41)	33	0.324	0.540
				34	0.393	0.592
				35	0.195	0.634
				37	0.277	0.659
				38	0.176	0.544
Chen et al. (2010) [18]	Chinese	8–16	302 (134/168)	33	0.496	0.391
				34	0.464	0.482
				35	0.491	0.454
				37	0.467	0.528

## Data Availability

Data supporting the findings of this study are available from the corresponding author upon reasonable request.

## Abbreviations

The following abbreviations are used in this manuscript:

CVM	Cervical vertebral maturation
Tooth 31	Left mandibular central incisor
Tooth 32	Left mandibular lateral incisor
Tooth 33	Left mandibular canine
Tooth 34	Left mandibular first premolar
Tooth 35	Left mandibular second premolar
Tooth 36	Left mandibular first molar
Tooth 37	Left mandibular second molar
